# Microneedle-based ocular drug delivery systems – recent advances and challenges

**DOI:** 10.3762/bjnano.13.98

**Published:** 2022-10-24

**Authors:** Piotr Gadziński, Anna Froelich, Monika Wojtyłko, Antoni Białek, Julia Krysztofiak, Tomasz Osmałek

**Affiliations:** 1 Chair and Department of Pharmaceutical Technology, Poznan University of Medical Sciences; 2 Student Research Group of Pharmaceutical Technology, Poznan University of Medical Sciences

**Keywords:** eye, microneedles, ocular drug delivery, ophthalmic drugs

## Abstract

Eye diseases and injuries constitute a significant clinical problem worldwide. Safe and effective delivery of drugs to the eye is challenging mostly due to the presence of ocular barriers and clearance mechanisms. In everyday practice, the traditional eye drops, gels and ointments are most often used. Unfortunately, they are usually not well tolerated by patients due to the need for frequent use as well as the discomfort during application. Therefore, novel drug delivery systems with improved biopharmaceutical properties are a subject of ongoing scientific investigations. Due to the developments in microtechnology, in recent years, there has been a remarkable advance in the development of microneedle-based systems as an alternative, non-invasive form for administering drugs to the eye. This review summarizes the latest achievements in the field of obtaining microneedle ocular patches. In the manuscript, the most important manufacturing technologies, microneedle classification, and the research studies related to ophthalmic application of microneedles are presented. Finally, the most important advantages and drawbacks, as well as potential challenges related to the unique anatomy and physiology of the eye are summarized and discussed.

## Review

### 1 Introduction

Since its first appearance in biomedicine, microtechnology is rapidly entering the world of pharmaceutical sciences, including pharmaceutical technology [1–4]. Due to the impressive evolution of new manufacturing techniques, it offers completely new opportunities to develop very sophisticated and precise drug delivery tools [5–6]. A large number of concepts and implemented projects, which is reflected in a large number of scientific papers, consistently pushes pharmaceutical technology to a new level of coping with various diseases, including those related to the eye [7–10].

Eye diseases and injuries are a major clinical problem worldwide, causing severe visual impairment or blindness in many millions of people [11–13]. According to World Health Organization, at least 2.2 billion people suffer from near or distance vision problems [14]. The most common factors that contribute to more or less severe vision loss include unaddressed refractive error, inflammation of the cornea, sclera and iris, conjunctivitis [15], dry eye syndrome [16], allergies [17], retinopathy [18–19], age-related macular degeneration [20], cataract [21], glaucoma [22], central retinal vein occlusion [23], and diabetic macular edema [24–25]. Depending on the prompt diagnosis and treatment, most of them can be completely or partially healed, but in the case of inappropriate or late therapy, irreversible changes may occur [26]. It is also worth mentioning the very high global annual costs of therapies reaching up to US$ 250 billion [14], though it has to be kept in mind that the incidence rate is closely related to such factors as gender, age, life quality, and socio-demographic index [27]. Most of the conditions mentioned require the use of pharmaceutical substances and their application in the surrounding of the eye, on its surface, or inside the eyeball. Also, in most cases, repeated drug applications for extended periods of time are required [28]. First of all, it should be taken into account that the eye is a very specialized sensory organ, separated from systemic circulation, with some distinctive pharmacodynamic and pharmacokinetic properties [29–30]. It is composed of various types of tissues including epithelia, connective tissue, smooth muscles, and vascular and neural network [31–32]. Undoubtedly, no other organ of the body, beside the skin, is so readily available or so easy to observe. However, due to the unique properties, there are specific opportunities but also difficulties in administering drugs to the eye [33]. Ophthalmic preparations represent an obvious alternative to the oral forms, which have many limitations such as low bioavailability due to hepatic circulation and potential food interactions, delayed onset of action, and systemic side-effects [34–36]. In addition, oral or intravenous administration require the use of higher doses in order to achieve the appropriate concentration of drugs in the area of the eyeball [37].

Taking into consideration the unique anatomy of the eye and the challenges related to drug delivery, a few important obstacles can be distinguished. Among the most important ones, physiological processes such as blinking and nasolacrimal drainage, anatomical barriers, efflux pumps, and metabolism in ocular tissues are responsible for drug elimination [38]. It is noteworthy that the tear film is completely replaced with a new one by the tear fluid secreted at a rate of 1.2 mL/min. The eye is also covered with a layer of mucin, which prevents exogenous substances from permeating to the deeper tissues. In the anterior segments of the eye, a few static barriers can be distinguished. The cornea (the corneal thickness is about 0.5 mm [39]) is covered with an epithelium layer consisting of 5–6 layers of closely packed cells equipped with tight junctions. Its thickness is approximately 50 μm [40], and it plays an important protective role. The posterior segment of the eye contains sclera, choroid, Bruch’s membrane, and blood–retinal barrier, which further prevent drug permeation. The thickness of the sclera, a membrane composed of randomly scattered collagen fibers, ranges from 0.5 to 1 mm, depending on the region of occurrence [41]. While the sclera is another barrier preventing drug permeation, the choroid is responsible for drug elimination. The blood–retinal barrier is connected to the retinal vascular endothelium with tight junctions hampering the permeation of active ingredients to the intraocular area. When designing non-invasive ophthalmic drug dosage forms, the main aim is to improve the bioavailability by increasing the diffusion across sclera, cornea, and conjunctiva [42]. In the case of externally administered drugs, rate and degree of absorption depend on the time the drug remains at the application site [43]. Up to 95% of the eye surface is covered by the sclera, which is well permeable to substances smaller than 70 kDa, including neuroprotective, antioxidant, or anti-angiogenic agents. For comparison, the cornea permeates substances with a mass not greater than 1 kDa [28]. Unfortunately, transscleral absorption is often reduced by elimination via nasolacrimal drainage pathways, tear protein binding, or drug metabolism [44]. The treatment efficiency is also decreased by constant movement of the eyeball and eyelids, and irrigation with tear fluid [45–46]. For these reasons, in addition to the search for new active pharmaceutical ingredients (APIs), novel technologically advanced ophthalmic drug delivery systems are being developed each year [38,47–48].

Among the ophthalmic preparations, the most commonly used are eye drops [49–51], ointments [52–53], or gels [54–55], containing the drug in a dissolved or suspended form [56–57]. They are applied by medical personnel or by the patient himself to the surface of the eye, to the conjunctival sac, or on the eyelid. Their main disadvantage is the need for frequent dosing, which can be troublesome. Hence, the treatment regimen is rarely followed, leading to a reduction in the effectiveness of the therapy [58–59]. Therefore, apart from designing the vehicle/base composition, drugs are often incorporated into appropriate carriers or introduced into systems whose purpose is to provide the expected concentration in the treated tissue for the desired time period. The most frequently studied and described are liposomes [60–61], micelles [60,62], microparticles [63–65], nanoparticles [66–67], micro- [68–69], and nanoemulsions [70–71]. Unfortunately, it has to be noticed that most technological solutions on the nanoscale are only promising at the laboratory stage. The transfer of such technologies to an industrial scale is often complicated and causes many difficulties [72–74].

Ophthalmic drugs can be also administered in the form of inserts. They are mostly solid or semi-solid forms with the appropriate size and shape, intended to be placed in the conjunctival sac. They consist of an active substance reservoir with a matrix structure or a film that regulates the rate of release of the API. Inserts are used less frequently because their application is difficult, they may cause visual disturbances, and the feeling of the presence of a foreign body in the eye causing discomfort of the patients [75–76]. Inserts can be divided into soluble (biodegradable or bioerodible) and insoluble (therapeutic systems) [77–78]. The advantage of soluble inserts is that they do not have to be removed from the eye. The rate of drug release is influenced by dissolution or erosion of the polymer matrix. Ophthalmic therapeutic systems belong to the group of non-biodegradable inserts from which the drug substance is released by diffusion at a constant controlled rate according to zero-order kinetics [79–81]. Drug-loaded soft contact lenses can be classified as non-dissolving implants [82–83]. Satisfactory results are also obtained with in situ gelling liquid implants [84–85] or film forming liquids [86–88].

In addition to the non-invasive methods mentioned above, there is a number of invasive techniques for the administration of ophthalmic drugs, mostly by injection into the vitreous or sub-surface parts of the eye. Subconjunctival and retrobular injections can provide rapid or prolonged release, depending on the composition of the formulation. The most common side effects in this case include local toxicity, tissue damage, eyeball perforation, optic nerve injury, occlusion of central retinal artery or vein, direct retinal toxicity during accidental puncture of the muscles, just to mention the most important [89–90]. In contrast, intravitreal injection is defined as a short and painless procedure, performed under local (drip) anesthesia on an outpatient basis [91]. It is worth mentioning that, after the injection, it is not possible to stop the action of the drug when side or toxic effects occur, among which retinal inflammation is the most commonly described. In order to minimize tissue damage, reduce the disruption of the membrane continuity, eliminate the risk of pathogens infections, ensure faster regeneration and improve the overall safety, the tendency to minimize the size of the needles became a significant trend and led to introduction of microneedles [92–98]. Taking into account the rapid progress in production technologies, it is already possible to obtain needles with a length of less than 1 mm, however, it can be expected that nanometer-sized needles will soon appear in use and revolutionize the treatment of ophthalmic diseases [99]. However, due to the very small size, injections with the use of microneedles require properly trained specialists as well as the use of advanced equipment [95,100].

Simultaneously with the development of single-microneedle technologies, research is focused on microneedle systems/patches for ocular drug delivery. Initially, such arrays were considered as painless, non-invasive, and highly efficient alternative for transdermal, intradermal, and percutaneous delivery of drugs [8,101–102]. Recently, it has been found that they can be successfully applied to the cornea or sclera [103]. Microneedles in skin administration have been studied for many years, including delivery of various therapeutic agents. These included antibiotics [104–105] or antifungals [106] for the treatment of local skin infections but also other drugs intended to achieve systemic action, for example, non-steroidal anti-inflammatory drugs (NSAIDs) [107], antihypertensives [108], and lipid-lowering drugs [109]. Moreover, microneedles (MNs) are broadly investigated as very promising platforms for the delivery of large molecules such as insulin [110] or nucleic acids [111–112]. Another application for which MNs have great potential is vaccine delivery [113–116]. The use of MNs in ophthalmology can still be considered as a fledgling area, however, with great potential but also many unknowns. The presented review summarizes the current data on ophthalmic microneedle patches, their manufacturing techniques, and the obtained therapeutic efficacy. In addition to a number of advantages, the questionable aspects related to this dosage form are also discussed.

### 2 Types of microneedle: materials, fabrication and properties for drug delivery

#### 2.1 Microneedle system types and properties

**Figure 1:**
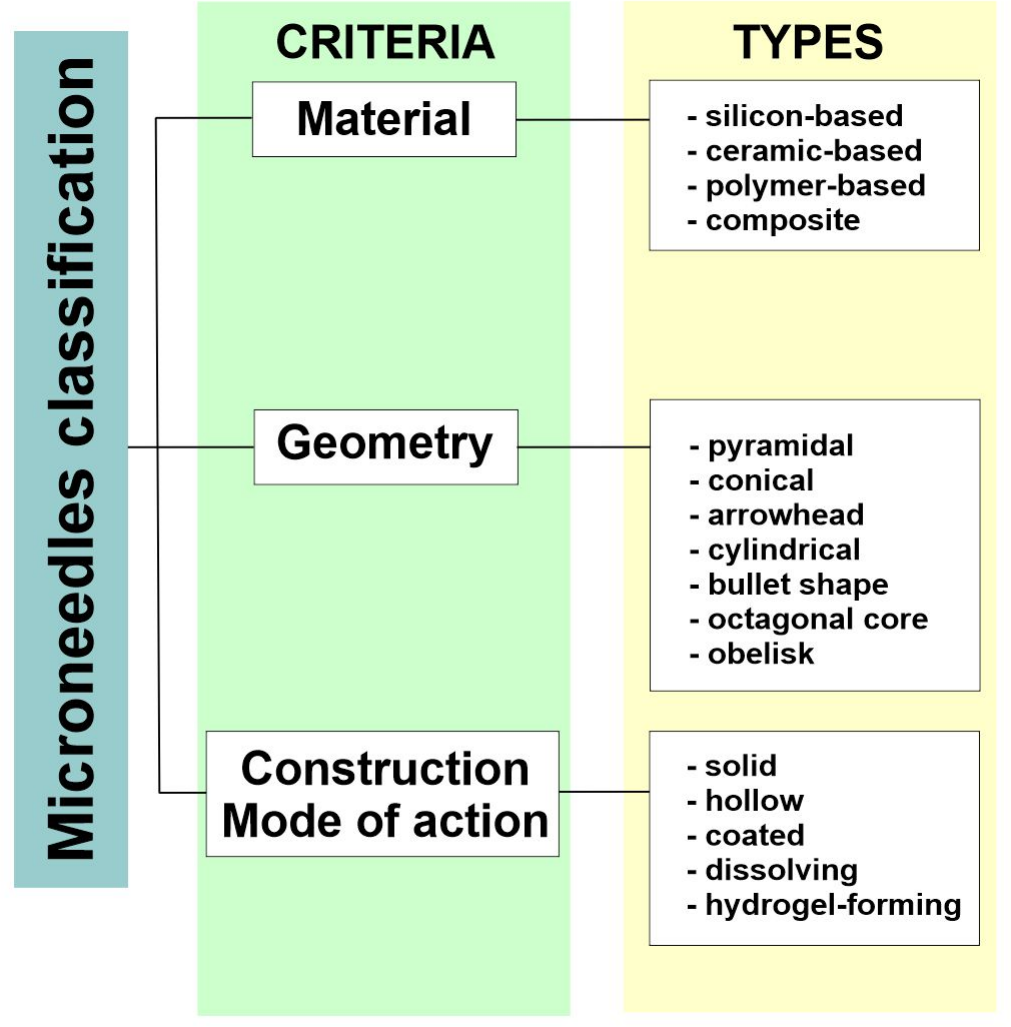
Microneedle classification criteria.

Microneedles applied for drug and vaccine delivery, as well as in diagnostics, can be classified according to several different criteria (Figure 1). The most common microneedle typifications are based on the geometry, the material applied to obtain the systems, the method of fabrication, the drug loading technique, and the mode of drug delivery [117]. Concerning the materials used to manufacture the microneedle arrays, the range of available substances is wide. The first material employed for this purpose was silicon. It is important to note that it offers versatile properties and the ability to form different microneedle geometries, which can be considered as an advantage. However, the manufacturing process can be complicated and the material is relatively expensive. Also, silicon is a brittle, non-compatible, and non-biodegradable material, which may cause skin irritation if the needles break and are deposited in the tissue [117–118]. Other non-degradable materials utilized to fabricate microneedles, include metals such as stainless steel [119] and titanium [120], ceramics, such as aluminum oxide [121], or synthetic polymers comprising polyvinylpyrrolidone (PVP) [122], polyvinyl alcohol (PVA) [123], and polymethacrylates [118,124–125]. Among the biodegradable materials, carbohydrates, including maltose [126], trehalose [127], and sucrose [128], are frequently mentioned. Moreover, biodegradable polymers such as poly(lactic acid) (PLA) [129], poly(glycolic acid) (PGA) [130], and poly(lactic-*co*-glycolic)acid (PLGA) [131] are widely investigated as microneedle materials. Among them, there are hydrogel-forming agents swelling upon the contact with interstitial fluid in the skin during microneedle application. These polymers include poly(ethylene glycol) diacrylate (PEGDA) [132] and poly(acrylic-co-maleic) acid (PAMA) [133]. It is also important to notice that there are numerous studies describing the use of composite materials containing combinations of various substances, both organic and inorganic. For example, studies involving PLA and carbon nanotubes [134], calcium sulfate and gelatin [135], gelatin and hydroxyapatite [136], and PLGA microparticles combined with PLA [137] are available in the scientific literature.

**Figure 2:**
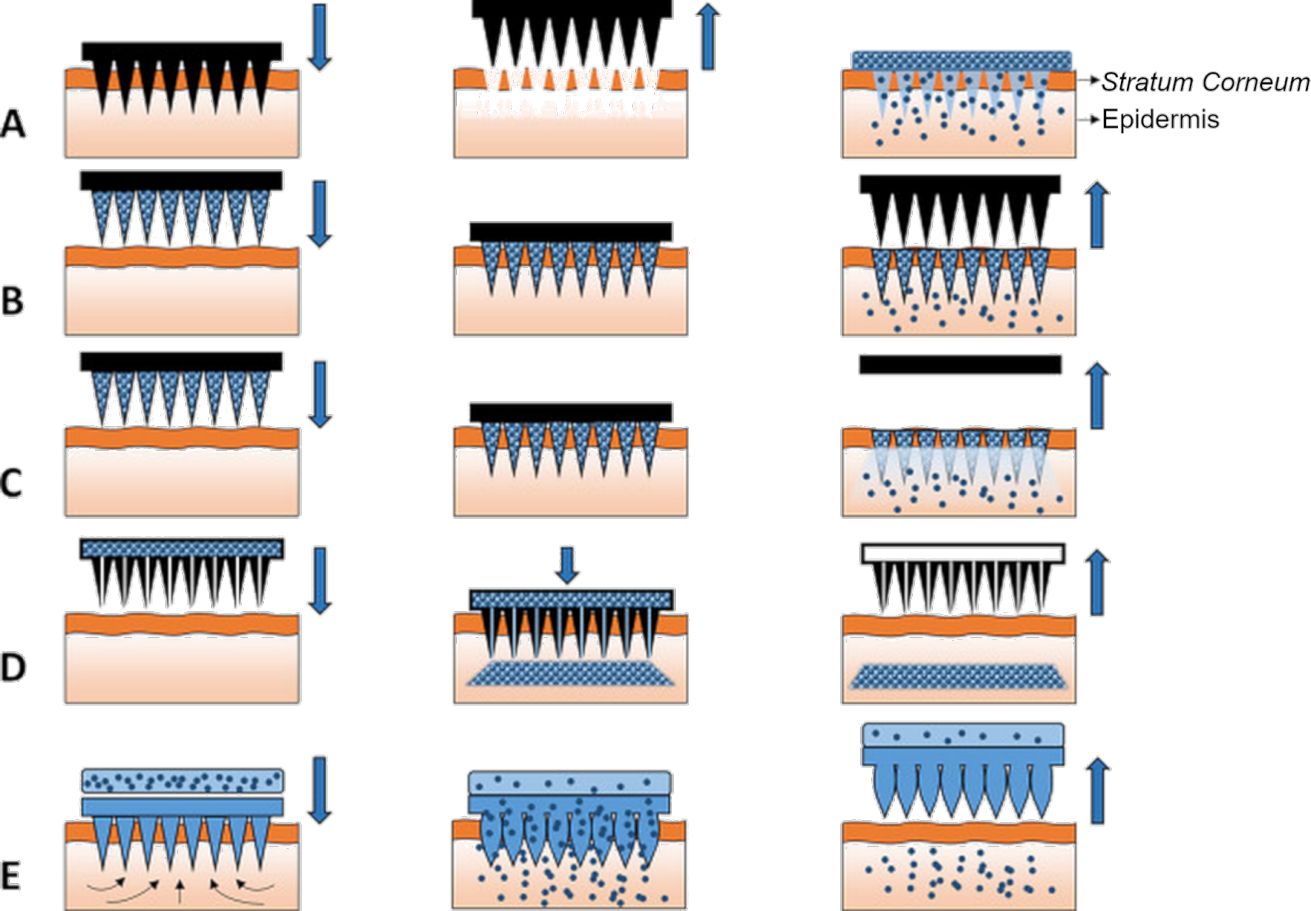
A schematic representation of five different MN types used to facilitate transdermal drug delivery. (A) Solid MNs for increasing the permeability of a drug formulation by creating microholes across the skin. (B) Coated MNs for rapid dissolution of the coated drug into the skin. (C) Dissolvable MNs for rapid or controlled release of the drug incorporated within the MNs. (D) Hollow MNs used to puncture the skin and enable the release of a liquid drug following active infusion or diffusion of the formulation through the needle bores. (E) Hydrogel-forming MNs take up interstitial fluids from the tissue, inducing diffusion of the drug located in a patch through the swollen microprojections. Figure 2 was reproduced from [139] (© 2016 E. Larraneta et al., published by Elsevier B.V., distributed under the terms of the Creative Commons Attribution 4.0 International License, https://creativecommons.org/licenses/by/4.0/).

Taking into consideration the shape and geometry of microneedles, they can be categorized as pyramids, cones, arrowheads, cylinders, bullets, octagonal cones, or obelisks [117,138]. Another classification system describing microneedle types is related to their structure and the mode of action (Figure 2).

Solid microneedles are usually investigated in potential dermal drug delivery for skin pretreatment. The systems are used to perforate the epidermis layer and to form channels allowing for better drug permeation to deeper skin layers. In this way, the active ingredient may act locally or reach the capillary vessels in the dermis and enter systemic circulation [117]. A study performed by Wei-Ze et al. [140] revealed that microneedle geometry was important in terms of enhancement of drug permeation across the skin. It was shown that the needles with flat tips were more efficient than the ones with sharp endings. Also, the system efficiency did not correlate with the number of microneedles. Coated microneedles are similar to the previously described type but they have an additional drug-loaded layer at the surface. The active ingredient is deposited in the tissue pierced with the microneedles during the application. It is noteworthy that, in the case of these systems, the drug-loading capacity is usually low. The active layer can be obtained through dipping or spraying with the drug solution, which is usually obtained as a water-based formulation containing also surfactants, thickening agents, and stabilizers. These excipients are necessary to provide the desired properties of the coating layer [114]. Hollow microneedles are similar to conventional needles, with a channel located inside and a hole at the tip. These systems can be used to deliver liquid drug formulations to deeper skin layers, depending on the length of the needles [141]. They have higher drug incorporation capacity compared to the solid and coated systems. Moreover, as they are usually prepared from ceramic materials, silicon, or metal, they display higher stiffness than polymer-based systems. The risk of clogging the internal canals of the needles upon application is mentioned in the literature as a possible drawback of these systems [142]. Dissolving microneedles are intended to deliver active ingredients that are sensitive to heat, as these systems can be prepared from water-based polymer solutions at room temperature. The polymer dissolves in the tissue after the administration and the incorporated drug is released in this way. The dissolution time required to deliver the full amount of the drug is usually a few minutes; therefore, these microneedles are frequently designed to separate from the pedestal and remain in the tissue [114]. Among the most important disadvantages of dissolving microneedles, a low mechanical strength resulting in difficulties with piercing the tissues is mentioned. Some of the polymers employed in the manufacturing process are hygroscopic, which can also decrease the physical stability of the final product [143]. Other polymer-based microneedles are hydrogel-forming systems, which are obtained with the use of hydrophilic substance swelling upon the contact with the fluid at the administration site. It is noteworthy that the polymer matrix does not dissolve under physiological conditions and can be removed after the drug is released [117,144]. As the microneedles absorb interstitial fluid from the surrounding tissue, they can be utilized not only as drug delivery systems but also as minimally invasive diagnostic tools [145–146]. The advantages of hydrogel-forming systems include relatively high drug-loading capacity and the possibility to modify the drug release rate with respect to the individual needs, which is usually achieved through adjusting the polymer crosslinking ratio [147]. Moreover, the polymers usually employed in this type of formulation are biocompatible, which decreases the toxicity risk [144].

#### 2.2 Microneedle manufacturing methods

The selection of a manufacturing technique should take into account the intended use of the microneedle system and the material from which it is to be made. In addition, the technique must be adapted to the properties of the drug.

The most common materials used in the production of microneedles are silicon, ceramics, and metals, such as stainless steel and titanium. Also, biodegradable polymers such as poly(lactic acid), poly(glycolic acid), and non-biodegradable polymers, for example, photolithographic epoxy resins are used [148]. The methods used for the production of microneedles include lithographic or laser techniques, casting, and 3D printing, to mention a few. The laser cutting technique can be used to produce microneedles from metals or polymers. The main part of the process is cutting microneedles out of a plate with a laser and then bending them. The alignment of the tips can be achieved by electropolishing [149]. A similar technique is laser ablation. In this case, the substrate, absorbing the laser beam, heats up and evaporates or sublimes, which yields engraved 3D patterns [150].

In the fused deposition modelling (FDM) method, the thermoplastic material is heated to its softening point, then extruded through a nozzle and applied layer by layer to the build plate where it quickly solidifies [151]. In the microstereolithographic method, a prepared polymer or a mixture of polymers undergoes polymerization under the influence of a high-energy light source (e.g., UV radiation) [150]. Digital light processing (DLP) is also a technology based on photopolymerization of photosensitive polymers, but in this case each layer of the polymer is projected as whole [152].

A more complicated method is two‑photon polymerization (TPP), which uses a near-infrared beam instead of UV radiation. TPP initiates the polymerization of the resin by multiphoton absorption [153].

An alternative to methods using UV or heat is the droplet-born air blowing method (DAB). It is suitable for drug molecules that can be inactivated. In this method, polymer droplets are placed between two sheets. As the sheets are pulled apart, the droplets elongate and the resulting needle-shaped matrices are dried by the flowing air [154–155].

Another well-known method is photolithography. In this technique, a silicon wafer is covered with a photosensitive or photoresistant polymer. The plate is then exposed to UV radiation. A pattern is formed depending on the coverage of the photosensitive or photoresistant layer. The wafer is then etched. A distinction can be made between wet and dry etching. The wet etching process uses a potassium hydroxide solution, while dry etching includes the physical methods ion milling and sputtering and the chemical method high-pressure plasma [156]. Lithographic techniques can be also used to prepare molds for the micromolding method. Micromolding, also known as solvent casting, is quite popular due to cost-effectiveness and simplicity. This method uses a mold usually made of silicone. The prepared mold is filled with a polymer solution or mixture. Then, air voids are removed with a centrifuge or through vacuum and the mold is baked in the oven. After cooling down, the finished microneedles are removed from the matrix [151,156–157]. This type of method is chosen to obtain microneedles for ophthalmic use [158–160].

Methods of drug-loading depend on the type and construction of the manufactured microneedles, as well as the applied material. The properties of the drug must be carefully taken into consideration, as some active ingredients may decompose at higher temperatures or upon irradiation. In the case of solid microneedles prepared with the use of porous ceramic materials, the pores in the carrier material can be filled with the active ingredient in liquid or solid form. In the first case, the drug diffuses from the solution in the microneedle pores upon application. In the other case, the drug solution is loaded to the microneedles and in the next step the formulation is dried and the drug precipitates inside the pores. Upon application, the drug dissolves in a physiological fluid and, in this form, can permeate to the deeper tissues [161]. In the techniques involving polymers applied to obtain solid microneedles, the drug may be dissolved [162–163] or suspended [164] in the polymer or monomer solution. In the manufacturing of more complex systems, with the active ingredient incorporated in a specific compartment of a microneedle, more complex procedures involving both drug-loaded and drug-free solutions may be employed [165]. Hollow microneedles containing an empty canal inside are usually filled with active ingredient solution, either passively or with the use of pressure-driven methods [161]. In the systems using passive diffusion, the drug solution can be loaded to the canals inside the microneedles [141] or to an external compartment [166]. In pressure-driven systems, a reservoir with different pumping mechanisms is attached to the microneedle systems. Usually, manually operated syringes are reported [167–168], as well as micropumps [169] or actuator-equipped devices designed for self-injection [170].

**Figure 3:**
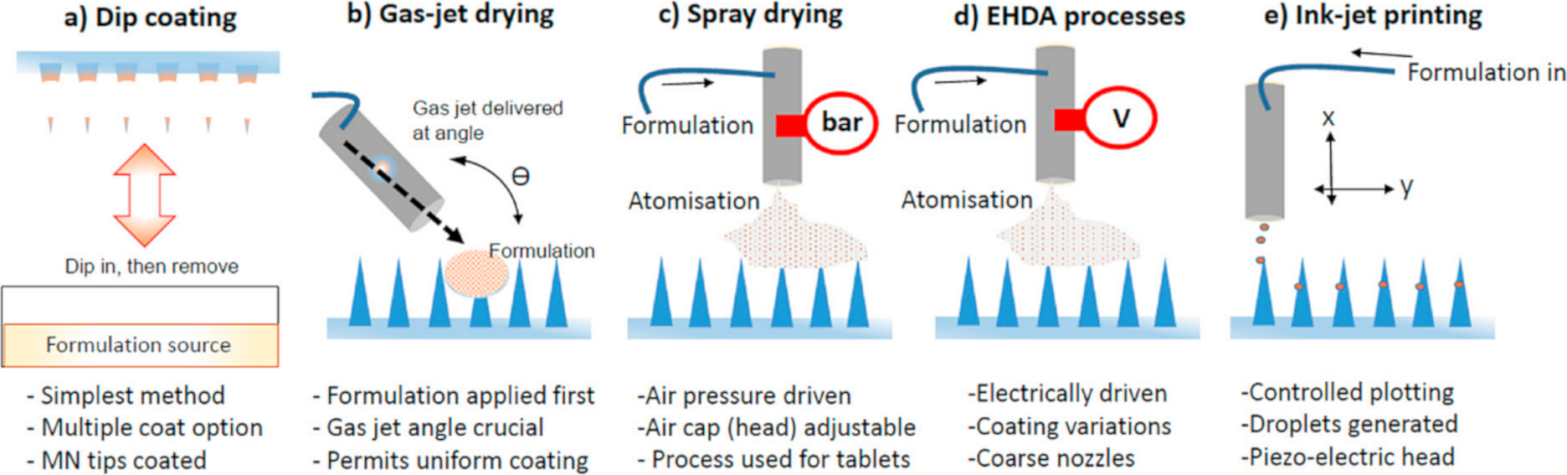
Illustrated examples of techniques used to coat MNs. (a) Dip coating. (b) Gas-jet drying. (c) Spray drying. (d) EHDA processes. (e) Ink-jet printing. Figure 3 was reproduced from [171] (© 2015 R. Haj-Ahmad et al., published by MDPI, distributed under the terms of the Creative Commons Attribution 4.0 International License, https://creativecommons.org/licenses/by/4.0/).

In coated microneedles the drug is deposited in the form of a thin layer on the external surface of the microneedle. Various coating techniques have been reported in the literature and summarized elsewhere [171–173]. The simplest method is dip coating, in which the microneedle array is immersed in the coating liquid and left to dry afterwards. The drying procedure can be accelerated with the aid of gas-jet drying. Another technique involves spray coating, which is technologically similar to the coating of oral solid dosage forms. Electrohydrodynamic atomisation (EHDA), in which electrically charged droplets are deposited on the microneedle surface, has been mentioned. As in piezoelectric inkjet printing, the droplets are released from the nozzle as a result of the application of an electrical field to a piezoelectric crystal, which distorts and pushes the liquid out. The most important techniques applied in microneedle coating are depicted in Figure 3 [171].

### 3 An overview of microneedle systems for ocular drug delivery

Considering the construction and the mechanism of action, four types of MNs used to deliver a drug to the eye can be distinguished in the scientific literature: (i) Solid MNs, used mostly for puncturing a biological membrane and forming pores, which are further used as canals for drug delivery. In the next step, MNs are removed and the pores are filled with a drug-loaded formulation. (ii) Drug-coated MNs with a drug-loaded layer deposited on the surface; the coating layer dissolves in physiological fluids upon administration and the active ingredient diffuses to the deeper regions. (iii) Dissolving MNs that contain the active ingredient dispersed in the MN matrix. The matrix is prepared with the use of soluble or biodegradable materials, and, upon insertion, the matrix-forming agent gradually dissolves or is hydrolyzed and the drug is released. (iv) Hollow MNs that contain an empty space inside. Usually, these devices are attached to a pressure-driven unit that pushes the drug-loaded liquid through the canal into the target site [174].

**Figure 4:**
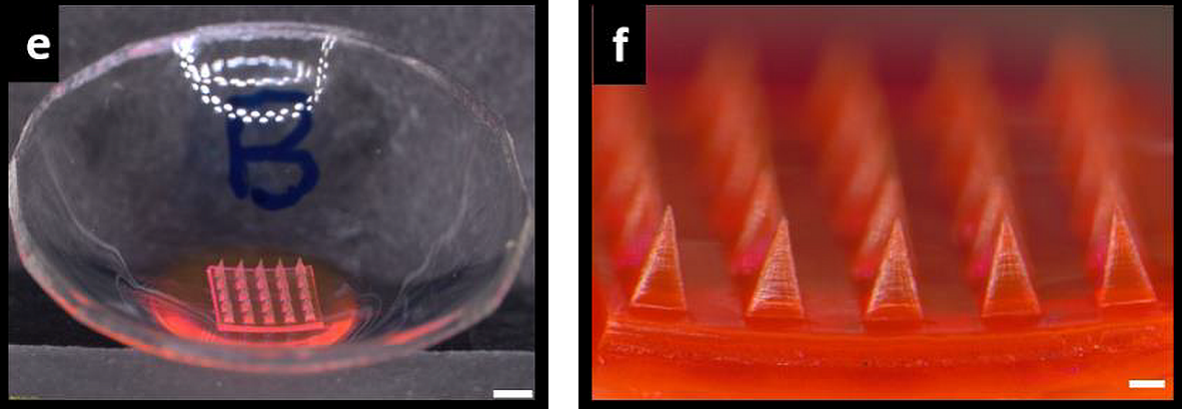
Stereomicroscopic image of the microneedle patch (e) and magnification of the microneedles (f), scalebars represent 1 mm and 200 µm respectively. Figure 4 was reproduced from [160] (“Microneedle ocular patch: fabrication, characterization, and ex-vivo evaluation using pilocarpine as model drug”, by G. Roy et al., Drug Development and Industrial Pharmacy, published on 11 Jun 2020 by Taylor & Francis Ltd), reprinted by permission of the publisher (Taylor & Francis Ltd, http://www.tandfonline.com). This content is not subject to CC BY 4.0.

All microneedle systems administered to the eye are currently at the stage of development and basic research, but the interest in them is systematically growing, which is reflected in numerous scientific and popular science publications. Microneedle ocular patches for the delivery of pilocarpine, a model drug usually employed in the treatment of glaucoma, was developed by Roy et al. The MN matrix consisted of PVA and PVP and contained 1 mg of the drug. The patches were fabricated by micromolding and mimicked the shape of commercial contact lenses (Figure 4). The patches were tested ex vivo with the use of excised human cornea and porcine eye globe. The amount of the drug that permeated across the cornea was distinctly higher from the microneedle system in comparison to the standard solution [160].

In a subsequent work, the authors presented two types of patches for the delivery of triamcinolone acetonide (TA), a model steroidal drug applied in numerous inflammatory conditions to the posterior segment of the eye. A microneedle scleral patch (MSP) and a microneedle corneal patch (MCP) were obtained by micromolding with the use of PVP. The ex vivo experiments performed on porcine eye globe showed that, in comparison to MCP and TA nanosuspension, MSP yielded much greater TA concentrations in the vitreous humor and choroid-retinal complex after 5 min of application. Moreover, the patches were tested with a rabbit eye model. In this case, after 24 h, significantly higher TA accumulation was observed for MSP in comparison to MCP and intravitreal injection [175].

**Figure 5:**
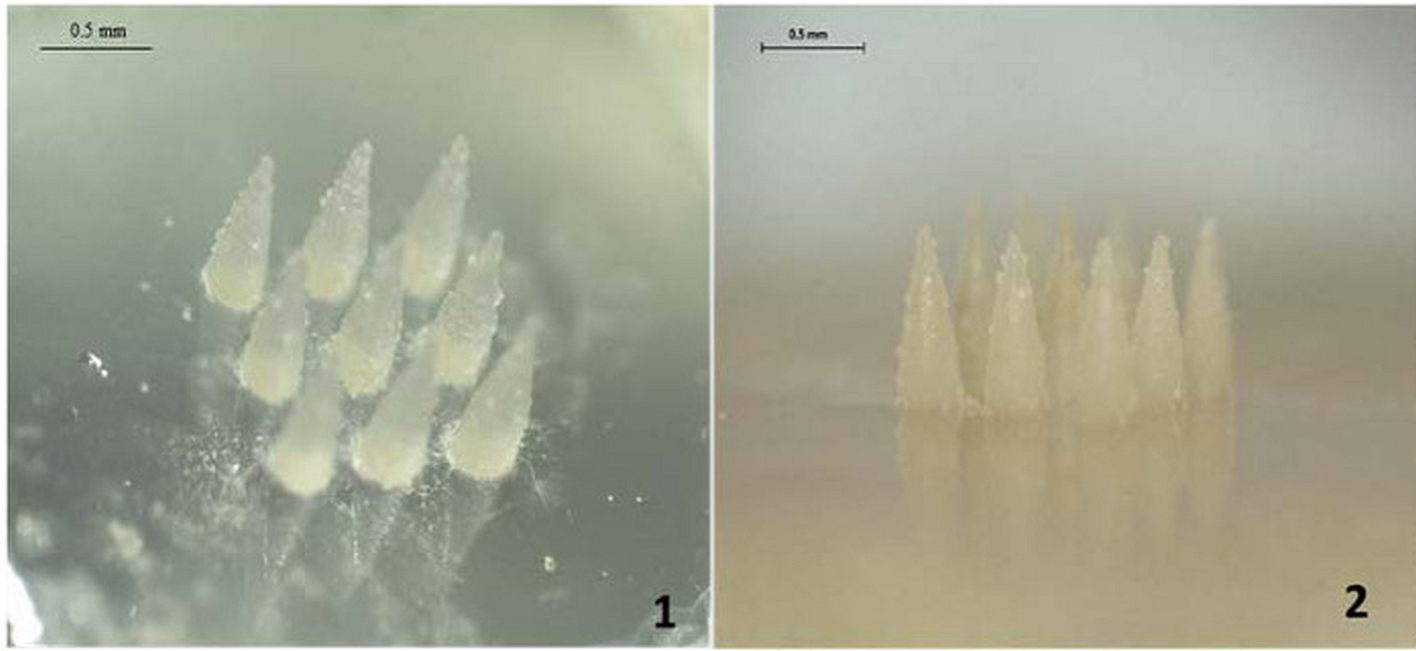
Microscopic image of dissolving MNs manufactured by Albadr and co-workers. Figure 5 was reproduced from [176] (© 2021 A. A. Albadr et al., distributed under the terms of the Creative Commons Attribution 4.0 International License, https://creativecommons.org/licenses/by/4.0/).

Albadr et al. designed a rapidly dissolving MN patch for ocular delivery of amphotericin B for the treatment of corneal fungal infections (Figure 5). The patch was manufactured from a blend of PVP and hyaluronic acid (HA) by molding. The components of the MN matrix ensured a very fast dissolution, approximately 30 s, in which the active substance was completely released, followed by a rapid onset of action. At the same time, it was emphasized that the manufacturing process did not reduce the antifungal activity of the drug [176].

**Figure 6:**
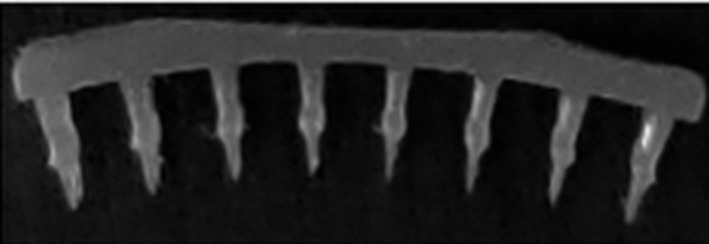
Microscopic image of the MNs obtained by Amer and Chen. Figure 6 was adapted from [178], M. Amer; R. K. Chen, "Hydrogel-Forming Microneedle Arrays for Sustained and Controlled Ocular Drug Delivery", Journal of Engineering and Science in Medical Diagnostics and Therapy, with permission from ASME. Copyright 2020 ASME. This content is not subject to CC BY 4.0.

Shi et al. proposed PLA/HA MNs for the ocular delivery of fluconazole, a widely known antifungal agent, employed in this formulation for potential keratitis treatment. As justification for the use of PLA, the authors indicated poor mechanical properties of HA and the associated risk of needle deformation during production and application. PLA served both as a scaffold to increase the matrix stability and to provide prolonged drug release. It was observed that the patches penetrated the rabbit corneal epidermis without irritation and, after removal, total recovery was observed after 12 h. The developed patch showed great potential as an alternative to ocular injection [177]. Amer and Chen fabricated PVA hydrogel-based microneedle arrays for the delivery of immunoglobin G1, a model protein resembling bevacizumab, a monoclonal antibody applied in the treatment of age-related macular degeneration (AMD) (Figure 6). First, the master mold was produced with the use of a light processing-based 3D printing technique. Then its shape was imprinted in the elastomer (Sylgard^®^ 184) and the prepared form was used to obtain the final microneedles by molding. The in vitro tests with the use of a Parafilm/polyethylene/nylon membrane equivalent and a fluid mimicking vitreous humor showed extended release of the active compound, compared to the rapid release after injection. The authors indicated that the MN arrays show a much more uniform drug release profile than the single injections [178].

As a continuation of the previous studies the authors created stimuli-sensitive bifunctional hydrogel microneedle arrays. In the first stage, after placing the patch on the eyeballs and piercing the membrane, the needles swelled and, as a result, wedged. Then, after the drug was released, the patch had to be irradiated by UV light (365 nm, 10 mW/m^2^) for 14 min with an OmniCure S2000 Spot Curing System (Excelitas Technologies, Waltham, MA) to cause the needles to shrink by about 20%, so they could be easily removed without the risk of damaging the eye. The MN matrix contained a mixture of PVA and spiropyran-conjugated *N*-isopropylacrylamide (NIPPAM) [179].

**Figure 7:**
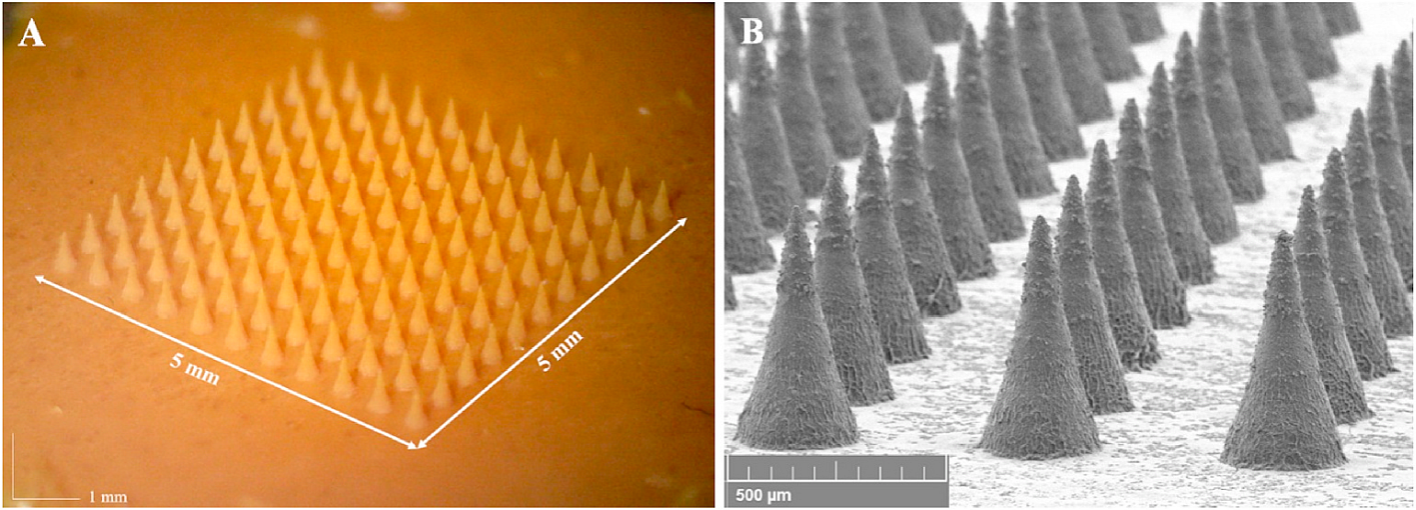
The physical appearance of 20.06% GAN + 5% HA + 1% FS under (A) digital microscope and (B) scanning electron microscope (×131). Figure 7 was reprinted from [180], Journal of Drug Delivery Science and Technology, vol. 61, by P. Suriyaamporn; P. Opanasopit; T. Ngawhirunpat; W. Rangsimawong, “Computer-aided rational design for optimally Gantrez® S-97 and hyaluronic acid-based dissolving microneedles as a potential ocular delivery system“, article no. 102319, Copyright (2021), with permission from Elsevier. This content is not subject to CC BY 4.0.

Suriyaamporn et al. employed a computer-aided design for the optimization of microneedle systems based on hyaluronic acid and a copolymer of methyl vinyl ether and maleic anhydride (Gantrez^®^S-97) produced by micromolding (Figure 7). Fluorescein sodium (FS) was used as a model compound with hydrophilic properties. Multiple parameters were evaluated, namely the physical and mechanical properties, ocular permeation, FS remaining in ocular tissue, dissolution time, insertion force, insertion depth, and ex vivo ocular drug delivery. The permeation studies on porcine eyeballs showed that, after application of the MN patches, the total amount of released dye was close to 18%, whereas from flat patches only 1% was released after 24 h [180].

**Figure 8:**
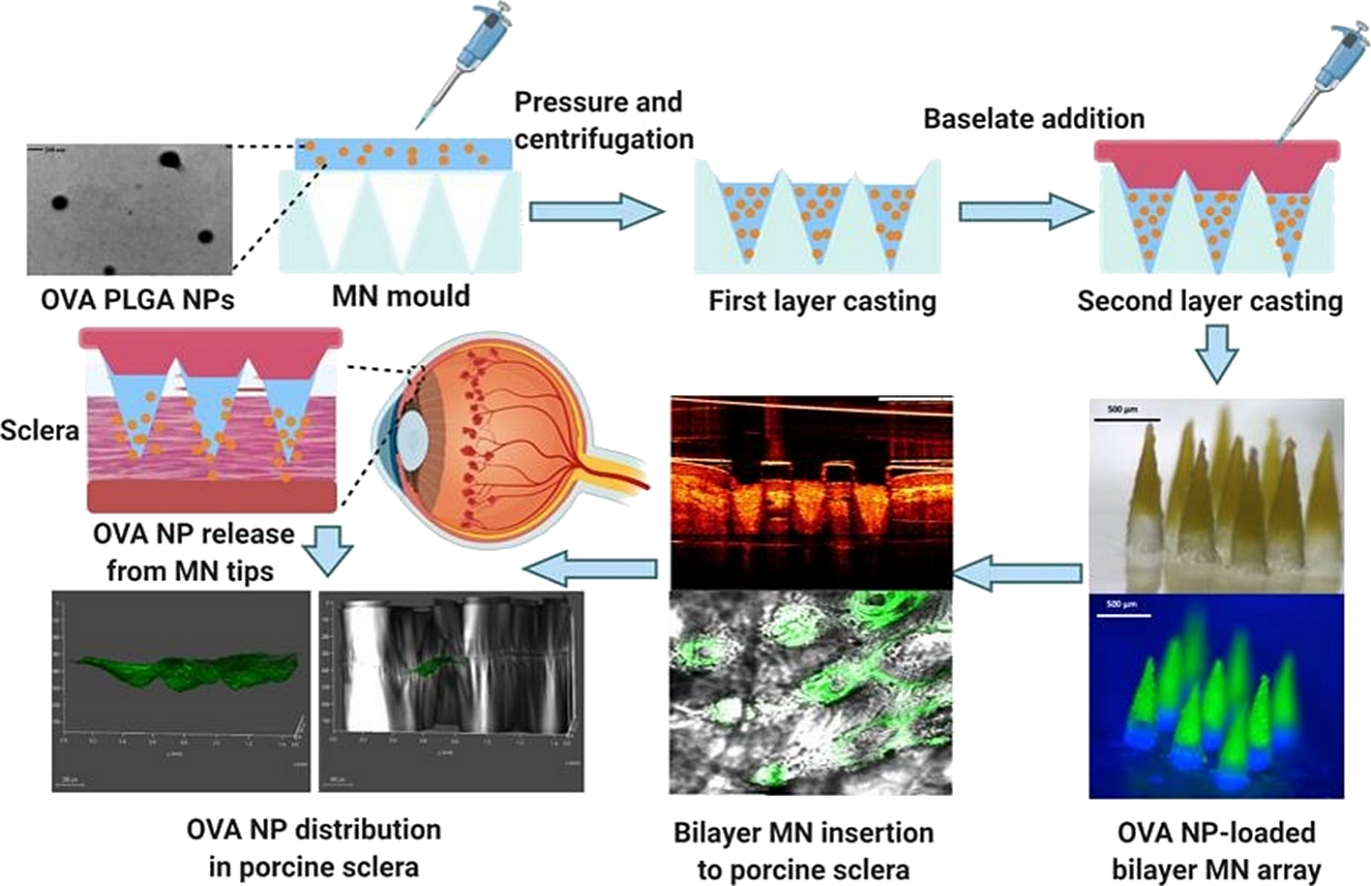
Individual steps in the production and use of microneedles containing nanoparticles developed by Wu and co-workers. Figure 8 was reprinted from [182], European Journal of Pharmaceutics and Biopharmaceutics, vol. 165, by Y. Wu; L. K. Vora; Y. Wang; M. F. Adrianto; I. A. Tekko; D. Waite; R. F. Donnelly; R. R. S. Thakur, “Long-acting nanoparticle-loaded bilayer microneedles for protein delivery to the posterior segment of the eye“, pages 306-318, Copyright (2021), with permission from Elsevier. This content is not subject to CC BY 4.0.

Li et al. fabricated PVP-based MNs for the ocular delivery of the insoluble drug brinzolamide, a carbonic anhydrase inhibitor applied in the treatment of glaucoma. The authors coated the microneedle matrix with a mixture of the drug with PVP and ethanol. Then, after evaporation of the solvent and solidification of the polymer with the drug, demolding was performed. In vivo studies on an animal model (rat) showed rapid in vitro drug release with 93% accumulative release at 2 h and a high corneal permeation of the drug [181]. Bhatnagar et al. employed micromolding techniques for the preparation of dissolving MNs for the corneal delivery of besifloxacin hydrochloride, a fluoroquinolone antibiotic useful in the treatment of bacterial infections. Due to blending of PVA and PVP, the obtained material revealed an appropriate mechanical strength to reach penetration depth up to 200 µm. The degree of drug penetration across human excised cornea after 24 h was assessed for the traditional suspension used for 5 min or 24 h and after 5 min of contact with the microneedles. The suspension used for 5 min was almost five times less effective than the MNs [158]. Nanoparticle-loaded bilayer dissolving microneedle arrays for the sustained delivery of proteins to the posterior region of the eye were developed by Wu and co-workers (Figure 8). Ovalbumin, a model protein, was encapsulated in PLGA-based nanoparticles by a water-in-oil-in-water double emulsion method. The nanoparticles were used to form microneedles in combination with various types of PVA. Then, after drying, a base layer made of an aqueous hydrogel was attached. It turned out that the MNs had adequate mechanical stability to puncture the sclera and then degraded very quickly releasing the nanoparticles (NPs) in less than 3 min. In turn, the slow disintegration of the NP-forming matrices resulted in the release of the active ingredient in a prolonged manner [182].

**Figure 9:**
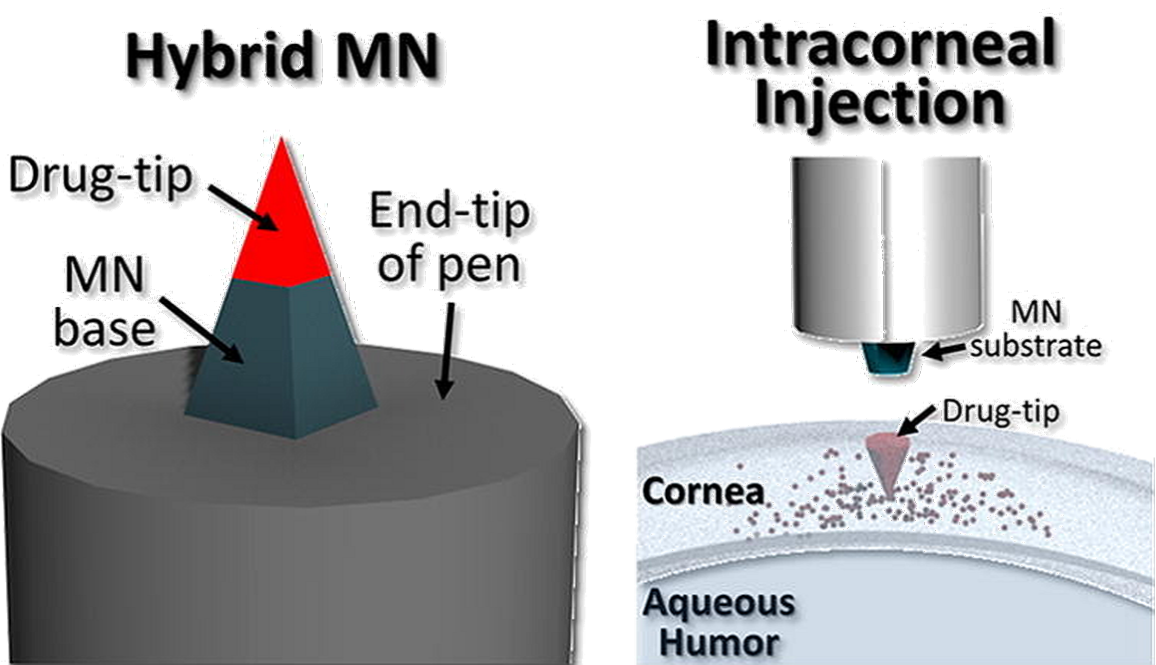
The hybrid detachable microneedle developed by Lee et al. Figure 9 was adapted from [183], Acta Biomaterialia, vol. 80, by K. Lee; H. B. Song; W. Cho; J. H. Kim; J. H. Kim; W. H. Ryu, “Intracorneal injection of a detachable hybrid microneedle for sustained drug delivery“, pages 48-57, Copyright (2018), with permission from Elsevier. This content is not subject to CC BY 4.0.

Lee et al. drew attention to the inconvenience of applying the MN patches to the surface of the eye, resulting from the constant movements of the eyeball and eyelid. The authors also pointed to the potential risk of hypoxia of the eye surface as a result of long presence of the patches on its surface necessary to assure the therapeutic effect. As a solution, the authors proposed administering the drug in the form of a single microneedle with detachable tip, which remains in the cornea for a certain period of time, ensuring sustained release (Figure 9). Two model drugs were used, namely FITC-dextran for the determination of release profiles and polyhexamethylene biguanide, an antimicrobial agent, for in vivo assessment of therapeutic efficiency. The drug-containing tip after optimization was delivered to the mouse cornea and efficiently reduced the progression of keratitis [183].

**Figure 10:**
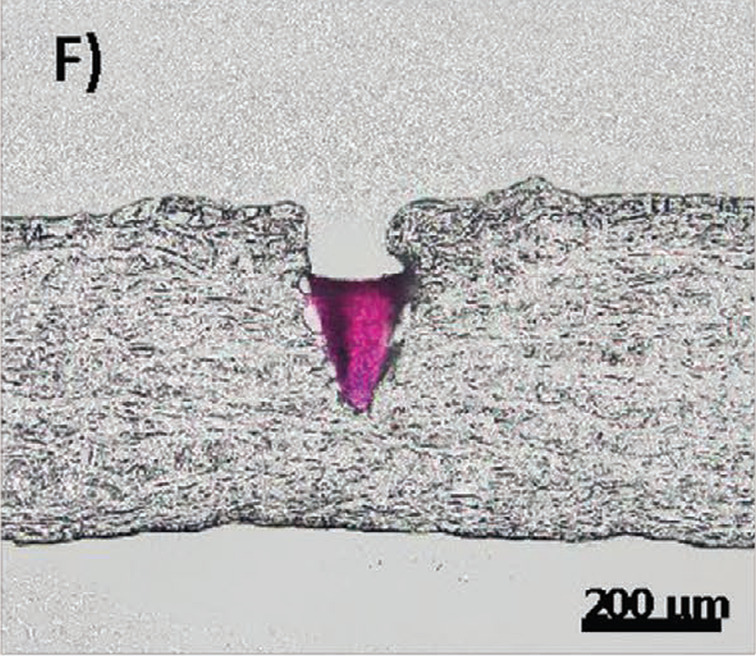
Cryo-sectioned optical image of a MN tip (stained with rhodamine B) embedded in the sclera by Lee et al. Figure 10 was reproduced from [184], Y. Lee et al., “Rapidly Detachable Microneedles Using Porous Water-Soluble Layer for Ocular Drug Delivery”, Advanced Materials Technologies, with permission from John Wiley and Sons. © 2020 WILEY-VCH Verlag GmbH & Co. KGaA, Weinheim. This content is not subject to CC BY 4.0.

The same group developed another rapidly detachable MN. The new idea was to modify the previously described concept by adding an additional fast dissolving layer separating the drug tip from the microneedle base. The layer consisted of a porous blend of PVA/PVP. Optimization of the composition and manufacturing process enabled almost immediate release of the tip upon contact with tear fluid and placing it at the appropriate depth applying manual pressure (Figure 10) [184].

**Figure 11:**
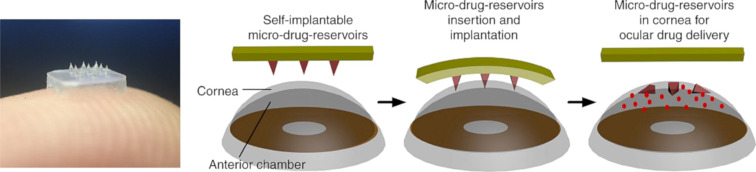
The photography and the principle of operation of patches developed by Than et al. Figure 11 was reproduced from [185] (© 2018 A. Than et al., distributed under the terms of the Creative Commons Attribution 4.0 International License, https://creativecommons.org/licenses/by/4.0/).

Than et al. fabricated corneal patches (2 × 2 mm) with self-implantable needle-shape microreservoirs (Figure 11). The microneedles were obtained by a simple micromolding method. The outer layer consisted of methacrylated hyaluronic acid (MeHA), while the interior was filled with unmodified HA. The needles were, in turn, attached to the HA patch. This design ensured the needles to detach immediately after application. Then, the inner layer of HA quickly degraded and the drug was released, while the MeHA shell acted as a depot form. Describing in vivo experiments with the use of animal (rat) corneal neovascularization as a disease model, the authors presented that delivering an anti-angiogenic monoclonal antibody (DC101) by such an eye patch caused a reduction of the area of neovascularization by about 90%. Moreover, due to the double compartment structure of the MNs, the rapid release of the anti-inflammatory compound (diclofenac) from the fast dissolving HA-based core provided a synergistic effect with the sustained release of DC101 from the outer layer. The authors also emphasized that it is possible to produce needles containing more than two compartments with different drugs [185].

In order to improve the transport of the steroidal drug difluprednate, applied in the treatment of anterior uveitis, to posterior regions of eye, Shelley et al. developed detachable two-component MN patches for extended release of the drug. The microneedles were made of Resomer, while the detachable layer was made of poly(acrylic acid) (PAA) [186]. This combination ensured that the needles were quickly inserted into the eye space followed by a slow release of the drug.

Datta et al. fabricated a lens-shaped MN patch of PVP for rapid corneal delivery of the macromolecular drug cyclosporine A (CsA) applied against uveitis, corneal injuries, vernal keratocojunctivitis, and other diseases with underlying inflammatory processes. Due to the composition, the MNs degraded and entirely dissolved within 60 s. The ex vivo experiments on pig excised cornea showed that, in comparison to Cyclomune eye drops, the application of the MNs resulted in higher values of drug flux and retention. CsA was evenly distributed within interior parts of the eye [187].

The presented studies indicate that the most important scientific research directions related to microneedles applied in ophthalmic drug delivery are focusing on hydrophilic matrices, mostly obtained with the use of PVP, PVA, and hyaluronic acid or its derivatives via molding techniques. It was shown that with these materials both suitable mechanical parameters and quick drug release can be obtained, which is crucial in terms of therapeutic efficacy and patient comfort. The obtained systems were either planar patches or were shaped into a contact lens, which provides ease of administration. It is also noteworthy that some of the presented dosage forms were rapidly dissolving microneedles and did not require prolonged contact with the eyeball surface thereby decreasing eye irritation and discomfort during application. However, this issue should be thoroughly investigated before releasing any microneedle array-based formulation to the pharmaceutical market. As can be concluded from the presented studies, the current state of knowledge is related mostly to physicochemical parameters and the number of studies performed with the use of in vivo models is rather limited. It is also noteworthy that the available data refer to animals and, to the best of our knowledge, no studies performed with human volunteers have been published so far.

### 4 Advantages and disadvantages of microneedle systems for ophthalmic drug delivery

Microneedle arrays as potential drug delivery systems offer numerous advantages, including ease of administration without professional assistance, the ability to overcome the external barriers of the human body decreasing the effectiveness of topical formulations, and minimal invasiveness. Ocular drug administration is usually associated with many challenges; poor bioavailability is among the most important ones [188]. Specific physiological conditions present in this body region are responsible for quick drug removal from the surface of the eyeball and for relatively short residence times of the formulation after administration. Reaching the posterior eye segment is even more difficult due to its poor accessibility and natural barriers of the eye [189]. According to the available scientific reports, microneedles offer some possible therapeutic improvements compared with the conventional formulations, which is accompanied by a better patient acceptability when classical intraocular injections are taken into consideration. However, it must be emphasized that dealing with this sensitive organ is usually associated with possible discomfort, even in the case of relatively soft formulations, like eye drops, gels and contact lenses. It is also important to note that even though the described microneedle systems are minimally invasive, there is a risk of infection and inflammation at the administration site, which may result in further discomfort and pain. One of the most appropriate directions seems to be the design of patches with easily detachable or dissolving microneedles. This is due to the fact that leaving the patch on the surface of the eye for a long time is certain to cause discomfort to patients. It is also extremely interesting to be able to produce such patches using 3D printing techniques, which repeatedly enable the introduction of appropriate doses of APIs, but also allow the amount to be adjusted to the requirements of a given disease and for a specific patient. In order to evaluate the actual relevance of the described systems, clinical trials involving human volunteers are crucial, as it was already mentioned by Dugam and co-workers [8]. However, to the best of our knowledge, all completed clinical studies related to microneedle-based formulations do not involve microneedle arrays but single microneedle devices [190]. It is obvious that further investigations are necessary to define the directions of formulation development and to find the most important limitations of these systems. The currently available reports describe mostly formulation studies and the results of in vivo tests employing animal models. It must be emphasized that the results are usually promising and show the potential for scientific and clinical development of microneedle arrays.

### 5 Future directions

As was shown in the reviewed studies, the advantages of microneedles as ocular drug delivery systems are indisputable, even though there are also some important drawbacks. Taking into consideration the materials and methods employed in the literature studies, it is obvious that the most important trends in this area focus on quickly dissolving systems obtained with the use of hydrophilic polymers. These formulations are intended for short residence times at the eye surface to minimize the discomfort and the risk of side effects, including irritation, tissue damage, infection, and inflammation. However, the data regarding the possible risk related to ophthalmic microneedles is still insufficient and this area requires further investigation. As most of the safety issues still need to be studied in detail, there are currently no attempts to introduce any microneedle-based ophthalmic formulation to the pharmaceutical market, except for those containing a single microneedle instead of microneedle arrays. However, some important lessons can be learned from the studies describing single microneedle injections. Depending on the number of parameters, including, for example, the injected liquid volume, the formulation can be perceived by the patient as painful or acceptable [98]. Therefore, all these factors must be carefully considered and optimized. As we already mentioned, there is also a need for randomized clinical studies using microneedle arrays.

Another important challenge related to ophthalmic drug delivery systems in general is sterility. In order to provide the drug stability but also the stability of the polymers applied to obtained system, an appropriate sterilization method must be selected and optimized. So far, this issue has not been properly addressed and thoroughly investigated, as can be concluded from the limited scientific literature on this subject.

## Conclusion

The treatment of ocular diseases, especially those localized in the deeper tissues, can be challenging due to the poor accessibility of eye tissues, as well as anatomical and physiological barriers present in the eyeball. The conventional therapeutic approaches, including topical formulations such as eye drops and gels, as well as systemic ones, frequently prove to be ineffective, which is related to poor bioavailability of the drug. As an alternative, direct injections into the eye tissues have been proposed. However, this approach is associated with significant side effects and poor acceptability by the patient due to the high invasiveness. Microneedles can be considered as a minimally invasive compromise between the topical formulations, which are acceptable but reveal poor effectiveness, and direct injections, which are more effective but invasive. With this novel approach, the active ingredient can be delivered to the target site with good precision and minimized risk of tissue damage, pain, and infection [98]. This article presents the most important difficulties that must be taken into consideration regarding ophthalmic drug delivery, as well as the current research directions explored in the field of MNs investigated as drug delivery systems. As it was already mentioned, the presented studies indicate that these novel systems reveal an enormous potential related mostly to their minimal invasiveness and the possibility to administer them without any professional assistance. These advantages are extremely important in terms of patient compliance and may significantly contribute to the improvement of treatment efficacy. Table 1 summarizes the most important information about the research described in this review.

**Table 1:** Summary table containing the most important information discussed in the review, sorted chronologically.

API/active agent (activity, condition treated)	MN system	Matrix composition	Fabrication technique	Effects/conclusions	Ref.

FITC-dextran (model drug) and polyhexamethylene biguanide (model antimicrobial)	detachable hybrid MN pen	MN base: SU-8 resin detachable tip: PLGA	premolding/ pressure-assisted transfer molding	drug containing tip after optimization was delivered to the mouse cornea and efficiently reduced progression of keratitis	[183]
immunoglobulin G (model antiagiogenic agent)	self- implantable microneedle patch	methacrylated HA	cross-linking, molding	due to the double compartment structure of the MNs the rapid release of the anti-inflammatory compound (diclofenac) from the fast dissolving HA-based core provided the synergistic effect with the sustained release of DC101 from the outer layer	[185]
besifloxacin HCl (antibiotic, bacterial infections)	hydrogel MNs	PVA/PVP	micromolding	sufficient mechanical strength to reach 200 µm penetration depth; MNs were much more efficient than traditional suspension	[158]
pilocarpine (reduction of intraocular pressure, glaucoma)	contact lens-shaped MN system	PVA/PVP	micromolding	better permeation across the cornea in vivo in comparison to drug solution	[160]
immunoglobulin G1 (model for bevacizumab molecule)	self-adhesive MN patch	PVA	micromolding with the use of 3D printed master mold	prolonged in vitro release up to 4 weeks in comparison to injection	[178]
—	rapidly detachable MN pen	MN base: SU-8 resin dissolving layer: PVA/PVP detachable tip: PLGA	micromolding	optimization of the composition and manufacturing process enabled almost immediate release of the tip upon contact with tear fluid	[184]
—	Photo- responsive hydrogel MN system	polyvinyl alcohol and spiropyran-conjugated N-isopropylacrylamide	micromolding	easy detachment of the patch after drug release due to the light-induced shrinkage of the matrix	[179]
ovalbumin encapsulated in NPs (model)	fast dissolving MN bilayer patch	PVA	molding, high speed centrifugation	rapid dissolution of MNs, less than 3 min	[182]
fluorescein sodium (model compound)	dissolving MNs	HA/Gantrez^®^ S-97	micromolding	permeation studies on porcine eyeballs showed that due to application of the MN patches the total amount of released dye was close to 18%, whereas from flat patches only 1% was determined after 24 h	[180]
brinzolamide (carbonic anhydrase inhibitor; glaucoma treatment)	dissolving MNs	PVP K90	casting/demolding	rapid in vitro drug release at 2 h, high corneal permeation	[181]
amphotericin B (antifungal)	rapid dissolving MN patch	PVP/HA	micromolding	degradation of the MN up to 30 s, fast onset of drug action	[176]
fluconazole (antifungal)	dissolving MN array patch	PLA/HA	micromolding	satisfactory drug intracorneal pentration with no irritation and tissue recovery up to 12 h	[177]
difluprednate (anti-inflammatory)	rapid dissolving MNs	poly(ᴅ,ʟ-lactide-co-glycolide)	micromolding	matrix diffusion-controlled release over the 7-day	[186]
triamcinolone acetonide (anti-inflammatory)	microneedle scleral patch	PVP	micromolding	greater safety score compared with intravitreal injection	[191]
cyclosporine A (immunosupressant, uveitis and other inflammatory conditions)	dissolving MNs	PVP	micromolding	completely dissolve in the cornea within 60 s, enhanced flux and retention of the drug	[187]
